# The Sclerotic Scatter Limbal Arc Is More Easily Elicited under Mesopic Rather Than Photopic Conditions

**DOI:** 10.1371/journal.pone.0150314

**Published:** 2016-03-10

**Authors:** Eric Denion, Anne-Laure Lux, Frédéric Mouriaux, Guillaume Béraud

**Affiliations:** 1 Department of Ophthalmology, CHU de Caen, Caen Cedex 9, France; 2 Inserm, U 1075 COMETE, Caen Cedex 9, France; 3 Medical School, Unicaen, pôle de formation et de recherche en santé, Caen Cedex, France; 4 Service d’ophtalmologie, CHU Rennes, Rennes, France; 5 Univ Rennes 1, Faculté de Médecine, Rennes, France; 6 Médecine Interne et Maladies Infectieuses, CHU de Poitiers, Poitiers, France; 7 EA2694, Université Droit et Santé Lille 2, Lille, France; 8 Interuniversity Institute for Biostatistics and statistical Bioinformatics, Hasselt University, Hasselt, Belgium; Save Sight Institute, AUSTRALIA

## Abstract

**Introduction:**

We aimed to determine the limbal lighting illuminance thresholds (LLITs) required to trigger perception of sclerotic scatter at the opposite non-illuminated limbus (i.e. perception of a light limbal scleral arc) under different levels of ambient lighting illuminance (ALI).

**Material and Methods:**

Twenty healthy volunteers were enrolled. The iris shade (light or dark) was graded by retrieving the median value of the pixels of a pre-determined zone of a gray-level iris photograph. Mean keratometry and central corneal pachymetry were recorded. Each subject was asked to lie down, and the ALI at eye level was set to mesopic values (10, 20, 40 lux), then photopic values (60, 80, 100, 150, 200 lux). For each ALI level, a light beam of gradually increasing illuminance was applied to the right temporal limbus until the LLIT was reached, i.e. the level required to produce the faint light arc that is characteristic of sclerotic scatter at the nasal limbus.

**Results:**

After log-log transformation, a linear relationship between the logarithm of ALI and the logarithm of the LLIT was found (p<0.001), a 10% increase in ALI being associated with an average increase in the LLIT of 28.9%. Higher keratometry values were associated with higher LLIT values (p = 0.008) under low ALI levels, but the coefficient of the interaction was very small, representing a very limited effect. Iris shade and central corneal thickness values were not significantly associated with the LLIT. We also developed a censored linear model for ALI values ≤ 40 lux, showing a linear relationship between ALI and the LLIT, in which the LLIT value was 34.4 times greater than the ALI value.

**Conclusion:**

Sclerotic scatter is more easily elicited under mesopic conditions than under photopic conditions and requires the LLIT value to be much higher than the ALI value, i.e. it requires extreme contrast.

## Introduction

**Fig 1:**
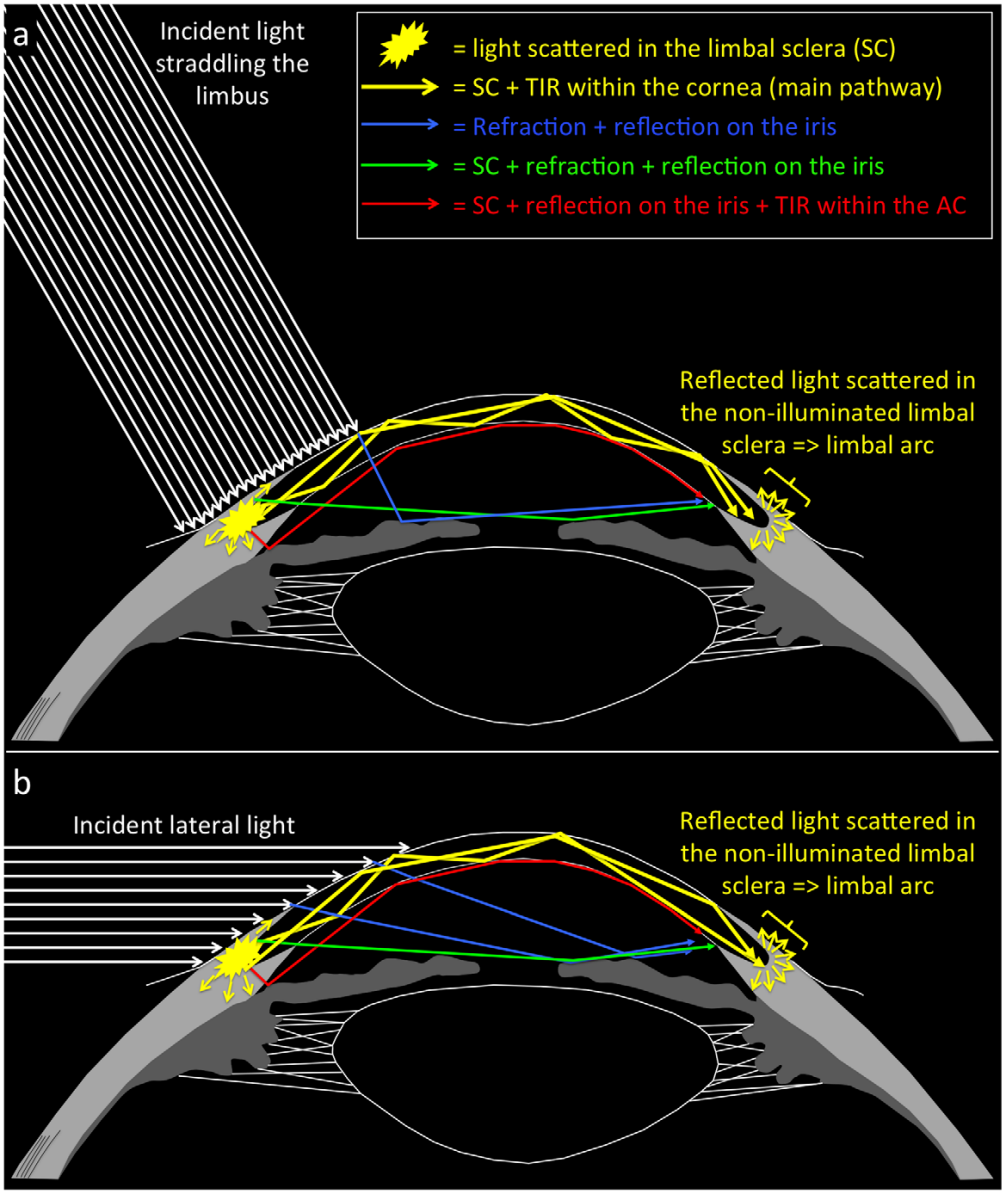
Schematic cross-section of a human eye depicting sclerotic scatter as (a) implemented in the present study, and (b) under natural conditions with a lateral lightsource. Some of the incident light straddling the limbus undergoes sclerotic scatter (SC), enters the cornea, undergoes total internal reflection (TIR) and reaches the other side, where it scatters a second time in the limbal sclera. Two different possible pathways for the light travelling through total internal reflection have been represented. Alternative ways for the incident light to eventually reach the opposite limbus include refraction through the cornea followed by reflection on the iris; SC followed by refraction through the cornea-aqueous humor interface followed by reflection on the iris; SC followed by reflection on the iris followed by TIR within the anterior chamber (AC).

Sclerotic scatter has been used for decades as a slit-lamp examination technique since its original description by Basil Graves in the 1930s [1]. It requires the slit-lamp light beam to be decentered on the limbus with central focusing of the biomicroscope [2,3]. The illuminated limbal sclera scatters light in all directions [1], some of which allow the light to enter the cornea, where it remains trapped due to total internal reflection [1] (Fig 1). This point of utmost importance, without which sclerotic scatter cannot be understood, was described and illustrated with unmatched clarity by Graves [1]. If the cornea is not clear, the light travelling through the cornea may strike opaque tissue, upon which it will be scattered and be seen by the clinician [1,4]. Sclerotic scatter is a sensitive technique that is effective for spotting corneal lesions [4] that may otherwise be overlooked. It easily reveals both the presence and the extent of corneal changes [5,6], however subtle these may be [3]. It does not, however, provide information as to the depth of the abnormality within the cornea and must therefore be combined with direct illumination with the slit beam [4].

**Fig 2:**
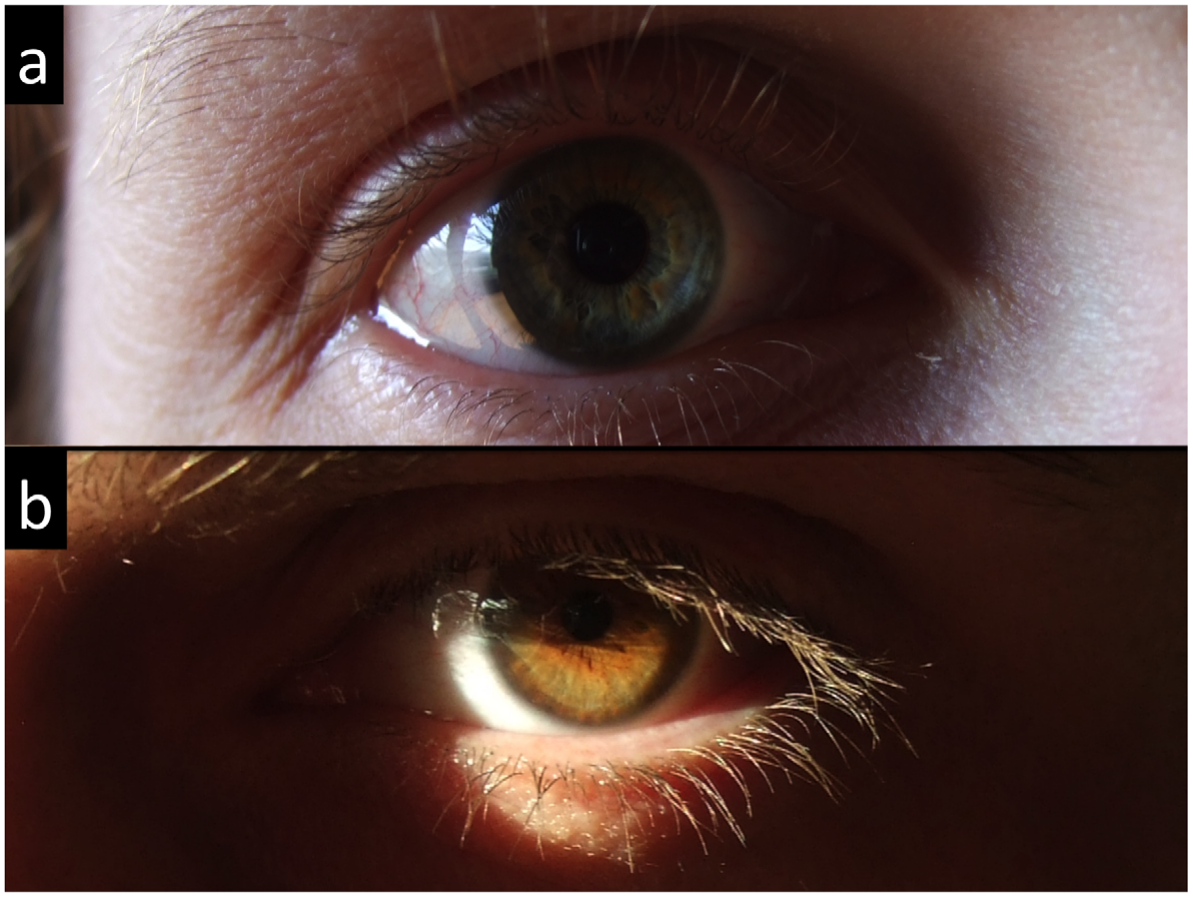
Sclerotic scatter in natural light conditions. a: In this right eye, the contrast is sufficient for the incident lateral light—striking the temporal limbus, travelling within the cornea to the opposite limbus through total internal reflection and scattering again at the nasal limbus—to create a faint yet visible scleral arc. b: In this left eye, the same phenomenon occurs, the incident light striking the nasal limbus and the scleral arc appearing at the temporal limbus.

A normal cornea is optically quiet, i.e. it will not reflect light along the viewing axis back to the examiner [1,2,4]. It will remain dark against an essentially dark background, allowing reflected light to travel unhindered from the illuminated limbus to the opposite, non-illuminated limbus, where it scatters a second time [1,4]. Some of this scattered light is directed back to the oculars and is visible to the clinician in the form of an illuminated scleral arc in the non-illuminated zone [1,4]. The posterior margin of the arc is projected in front of the supraciliary space, 0.5 mm behind the scleral spur [7], which may be useful for glaucoma surgery [7] or diode laser cycloablation. Other than these clinical applications in everyday life, we have frequently spotted this phenomenon in subjects whose temporal (or nasal) limbus is struck by a powerful lateral light while the nasal (or temporal) limbus is poorly illuminated. Under these circumstances the contrast is sufficient for the scattered light to appear as a faint illuminated arc [1] on the non-illuminated side (Figs 1b and 2). This observation prompted the present study, whose aim was to study the incident light illuminance levels on the limbus required to trigger perception of sclerotic scatter (faint light arc) in the opposite non-illuminated limbus, at different ambient light illuminance levels and in healthy volunteers. On spotting this phenomenon on several occasions, we had the impression that it was more easily elicited in participants with light irises. We therefore decided to investigate the possible role of iris shade on light arc eliciting in our study.

## Material and Methods

The data necessary to reproduce our analysis are freely available on a public figshare repository at http://dx.doi.org/10.6084/m9.figshare.1599798

This study was conducted on 20 healthy volunteers. The volunteers (medical students, fellows, nurses, orthoptists and secretaries) were all personally known to the authors. The study protocol and the consent procedure were declared and approved by the French Institutional Review Board of Caen University Hospital before the study began. The guidelines of the Declaration of Helsinki were followed, and written informed consent was obtained in each case.

**Fig 3:**
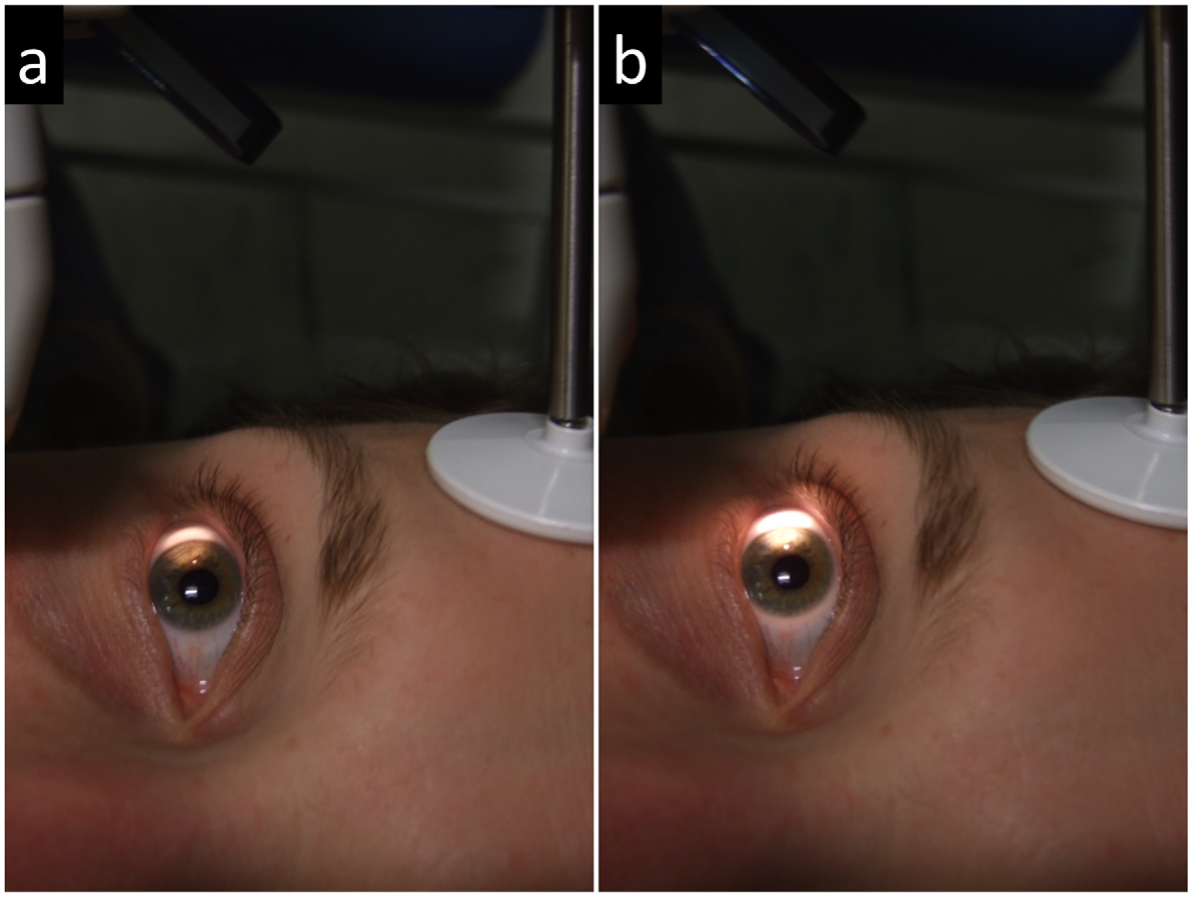
Determination of limbal lighting illuminance thresholds. The ambient light illuminance has been set at 40 lux. a: The lateral light illuminance is not sufficient to make the nasal scleral arc—characteristic of sclerotic scatter—appear. b: The lateral light illuminance is just sufficient (942 lux) to make a faint nasal scleral arc appear. This illuminance value is the threshold value.

Each of the volunteers was asked to lie down on his or her back, facing the ceiling, in a room fitted with adjustable neon lights. The ambient light illuminance (ALI), i.e. the illuminance at eye level, was first set to mesopic illuminance levels [8,9] (10, 20, 40 lux) and then to photopic illuminance levels [8,9] (60, 80, 100, 150, 200 lux) by adjusting the power of the neon lights and using a "CHY 630" luxmeter (IDDM+, Meaux, France). For each illuminance level, the temporal limbus of the right eye was illuminated with an 8 mm white lateral light spot straddling the 9 o'clock meridian, using a "BA 904" portable slit-lamp (Haag-Streit France, Chambéry, France) placed at a distance of 7 cm from the eye and at 30° from the sagittal plane (Fig 3a). The power of the light was increased until two independent examiners (ED, aged 40 and ALL aged 31) agreed that the nasal limbal light arc characteristic of sclerotic scatter could be discerned (Fig 3b).

Maintaining the slit lamp rheostat in the same position, the 8 mm spot was replaced with a 14 mm spot to fit the full measurement surface of the luxmeter, and the illuminance of this spot was measured at a distance of 7 cm from the luxmeter in a dark room. This illuminance—necessary and sufficient to trigger perception of sclerotic scatter for a given illuminance level—was called the "limbal lighting illuminance threshold" (LLIT). Strictly speaking, "sclerotic scatter" is a misnomer (or a metonymy) and is used to qualify the biomicroscopic technique aimed at examining and mapping corneal opacities. In the present study, "sclerotic scatter" does not refer to this technique, but solely to the scattering of light on the illuminated limbal sclera, then on the opposite limbal sclera (after the light has travelled through total internal reflection into the cornea).

In subjects 9, 10, 18, 19 and 20 the LLIT was measured a second time a few weeks after the first experiment in order to assess our measurement error. For each ALI, and for each of the 8 measurements, we called the LLIT value measured the first time *m1* and the LLIT value measured the second time *m2*. The measurement error was estimated by dividing the absolute value of half the difference between *m1* and *m2* by the median of *m1* and *m2*.

**Fig 4:**
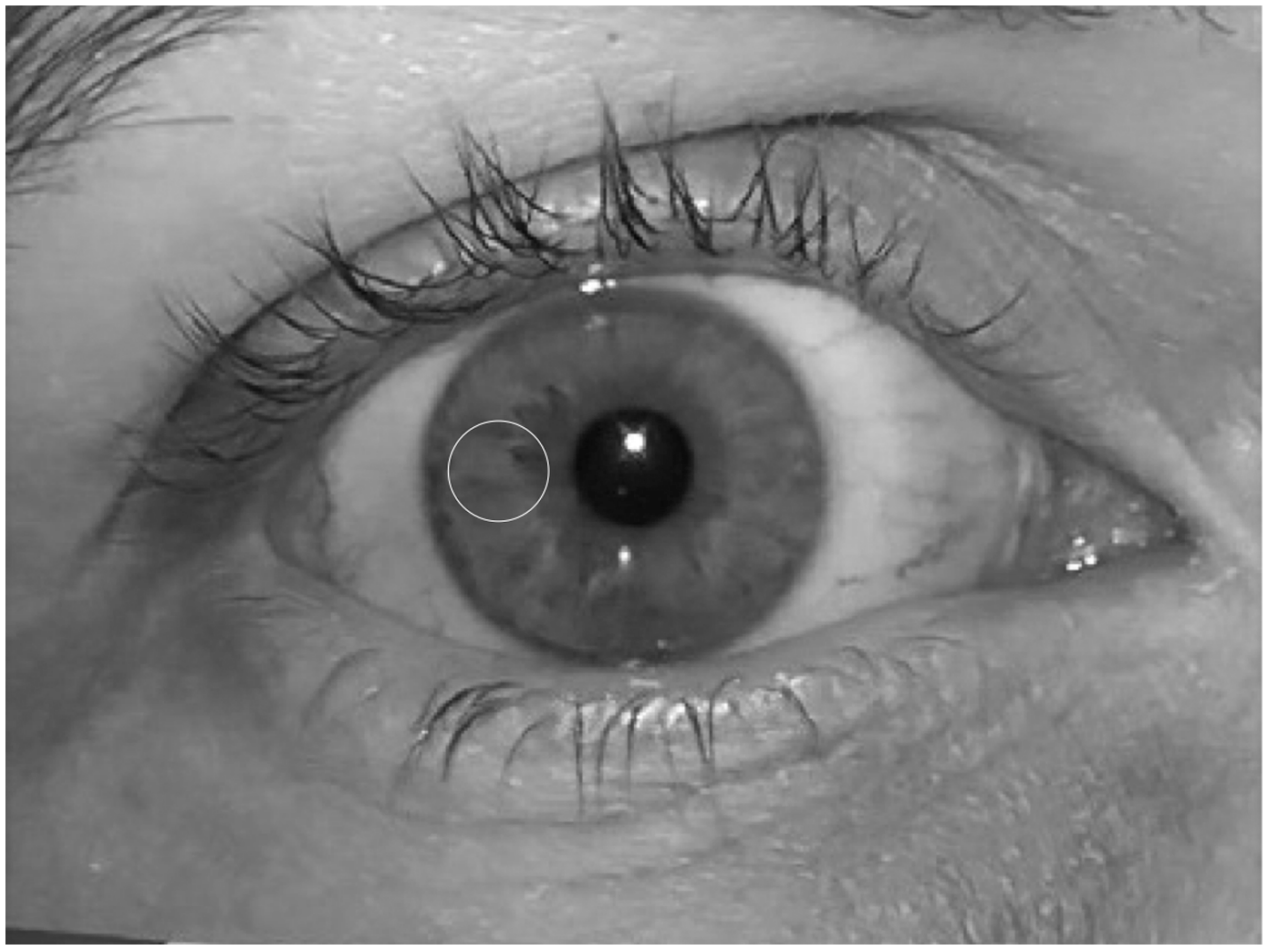
Iris-shade grading technique. Eye photograph showing the size and location of the circle used to obtain the median gray-level pixel value, the number allocated to each iris to characterize its shade.

To assess the possible influence of iris shade on the LLIT, we included 10 volunteers with light irises and 10 volunteers with dark irises. In an attempt to simply denote the iris shade, we allocated a number to each iris after implementing the following steps:

- For each healthy volunteer, a short film (".mov" format) of the right eye was made at a distance of 5 cm using a digital camcorder (HDC-SD80, Panasonic, Osaka, Japan) with the camcorder light switched on. At 5 cm, the illuminance provided by this light, measured with the "CHY 630" luxmeter, is 2000 lux.
- A ".pct" photograph was extracted from the film using the software QuickTime Player 7.6.6 (Apple Inc., Cupertino, California, USA).
- The ".pct" photograph was converted to ".jpg" format and into a gray-level picture using the software Photoshop Element 8.0 (Adobe Systems, San Jose, California, USA).
- This photograph was imported into Power Point for Mac, version 14.4.6 (Microsoft, Redmond, Washington, USA). A horizontal line segment was drawn from one limbus to the other through the pupil center using the line tool. A circle with a radius equal to a fourth of the line segment value—with its center on the horizontal line segment, midway between the temporal limbus and the temporal pupil border—was drawn using the circle tool (1/4 pt thickness). The horizontal line segment was erased and the slide converted to a ".jpg" picture (2999 x 1685 pixels; 72 dot per inch resolution).
- Using the magic wand tool of the software Photoshop CS5 (Adobe Systems, San Jose, California, USA), the area within the circle (Fig 4) was selected. Using the gray-level histogram-analysis tool, the median gray-level value was retrieved. This value was eventually assigned to the iris to characterize its shade.

We also wanted to know if the mean keratometry and corneal pachymetry values were correlated with the LLIT. We measured both of these parameters using a Tonoref II (Nidek, Maeham, Hiroishi, Gamagori, Aichi, Japan) and an EM-3000 (Tomey, Noritakeshinmachi, Nishi-Ku, Nagoya, Aichi, Japan) respectively.

### Statistical methodology

Generalized estimating equations (GEEs) [10] were used to model the association between the LLIT and ALI, iris shade, mean keratometry and central corneal pachymetry. The likelihood of a model is the probability that the model correctly predicts the outcome for a given set of values. Likelihood-based tools based have been developed and are widely used to assess and compare models. Information Criteria (AIC) and Bayesian Information Criteria (BIC) are the most popular of these tools, but cannot be applied to GEEs, which are based on quasi-likelihood. Recently, a Quasi-Likelihood Information Criteria (QIC) tool was developed as an adaptation of the AIC for GEEs [11]. Therefore, in our analysis, models were compared and assessed with backward selection using QIC. Similarly to AIC and BIC, the best model is determined by the lowest QIC. A log-log transformation was used on ALI and the LLIT, thus improving the model. Statistical analysis was conducted in R 3.0.0 [12] with the Geepack package [13].

## Results

The mean (standard deviation) age of the 20 volunteers was 30.5 (9.7) years; 5 were male. The mean (standard deviation) keratometry and central corneal pachymetry values were 43.5 (1.2) diopters and 535.8 (40.6) microns. The mean (sd/min-max) iris-shade gray levels for dark irises (10 subjects) and light irises (10 subjects) were 17.6 (4.3/13-28) and 52.3 (9.6/40-70) respectively.

**Fig 5:**
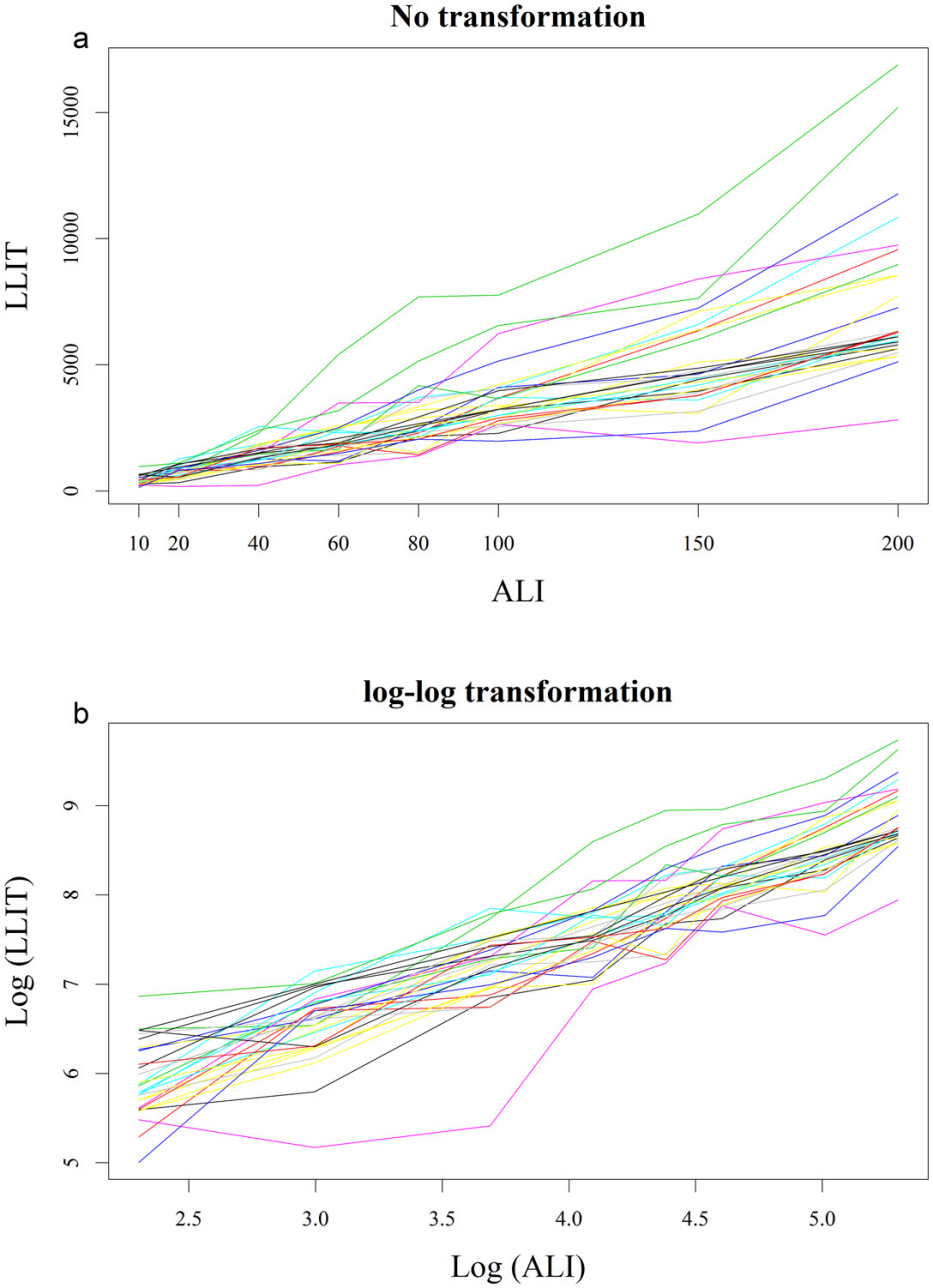
Plot of limbal lighting illuminance thresholds (LLITs) as a function of ambient light illuminance (ALI). a: without transformation, the relationship between the LLIT and ALI is non-linear, with a high dispersion of LLIT values for high ALI values. b: with log-log transformation, the relationship between the LLIT and ALI is linear.

The LLIT was proportional to ALI (Fig 5a). The highest values of ALI were clearly associated with a dispersion of the LLIT values (Fig 5a), showing a non-linear association, which required a log-log transformation to be appropriately modeled. Log-log transformation gave a better representation of the association (Fig 5b). We found a significant linear relationship between the logarithm of ALI and the logarithm of the LLIT (p<0.001) (Table 1). An increase in the ALI logarithm of one unit was associated with an average (standard error) increase in the log of the LLIT of 2.663 (0.637) or, in more practical terms, a 10% increase in ALI was associated with an average increase in the LLIT of 28.9% (more precisely, 100 x 1.10^2.663^–1%).

**Table 1:** Influence of the different variables on limbal lighting illuminance thresholds.

	Multivariate analysis (full model)		Multivariate analysis (final model)	
	Estimate (Std.err)	p	Estimate (Std.err)	p
log(ALI)	2.663 (0.637)	<0.001	2.663 (0.637)	<0.001
Mean keratometry	0.180 (0.061)	0.003	0.165 (0.062)	0.008
log(ALI) x Mean keratometry	-0.039 (0.014)	0.007	-0.039 (0.015)	0.007
Central corneal pachymetry	-0.001 (0.002)	0.7		
Repeated measurements	-0.057 (0.071)	0.42		
Light iris shade	0.161 (0.136)	0.24		

ALI, ambient lighting illuminance.

Neither iris shade nor central corneal pachymetry had an influence on the LLIT (Table 1). Mean keratometry values had a positive and significant influence, though very slight, on the LLIT, resulting in higher LLIT for high keratometry values under the same given ALI, at least for the lowest ALI. We also observed an interaction of mean keratometry values with the ALI (p = 0.007) (Table 1), with a negative (and minimal) coefficient. Accordingly, a high keratometry value decreased the effect of the ALI on the LLIT, resulting in lower LLIT values with increasing ALI values. For high ALI, this effect was predominant. However, the coefficients of both the keratometry and the keratometry-ALI interaction were far smaller than other coefficients therefore representing a very limited effect.

**Fig 6:**
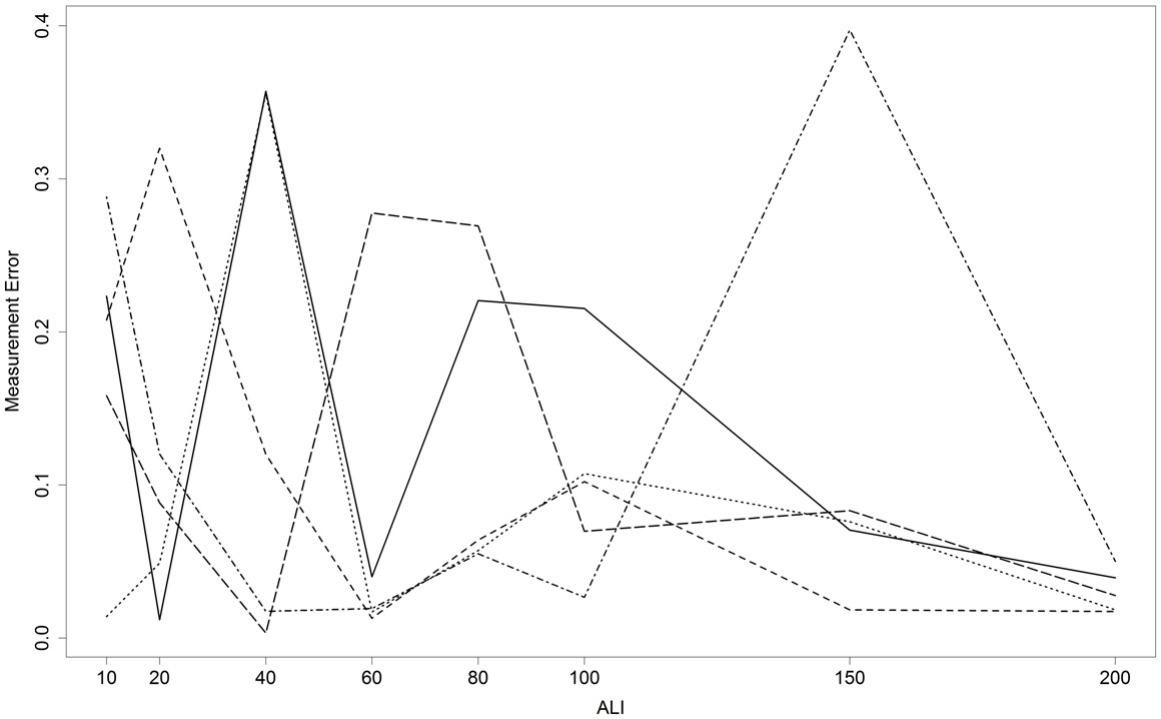
Plot of measurement error as a function of ambient light illuminance (ALI) in the 5 subjects in whom the lateral light illumination threshold was evaluated twice. The plot shows no particular relationship between measurement error and ALI. For illustrative purposes, we also developed a censored model, taking only ALI values of ≤ 40 lux into account. The likelihood of this linear model without log-log transformation was inferior to that of the log-log model (QIC [11] far greater than that of the log-log model). Nonetheless, the censored linear model showed that the LLIT was significantly associated and proportional to ALI, with a coefficient of 34.4 (p<0.001), and that neither mean keratometry (p = 0.29), pachymetry (p = 0.59) or iris shade (p = 0.12) had any statistically significant effect.

As detailed in Table 1, the LLIT was mainly correlated to ALI, as well as very slightly to the keratometry reading. A GEE approach was used, since it is able to handle correlations between repeated observations in the same participants. The variable “repeated measurements” had no significant influence in the model, showing that there were no significant differences between the first and second measurements in subjects in whom the LLIT had been evaluated twice. Fig 6 shows a plot of measurement error for the 5 subjects in whom the LLIT was evaluated twice. The measurement error bears no particular relationship with ALI.

## Discussion

To the best of our knowledge, the present study is the first to study how much incident light must illuminate one limbus to elicit the perception of the sclerotic scatter arc on the opposite limbus, for different ambient light illuminance levels.

Our study shows that a 10% increase in ALI requires a 28.9% increase in LLIT to trigger perception of sclerotic scatter. In other words, sclerotic scatter perception is more easily elicited for low ALI levels, i.e. under mesopic conditions. For high ALI values, the LLIT values are much more dispersed. Given that the measurement error bore no particular relationship with ALI, this finding implies that, in the case of low ALI, there is little variation in the effectiveness of lateral light transmission by the eyes of the various subjects, resulting in little variation in the LLIT. However, in the case of high ALI, the lateral light seems to be transmitted more efficiently in eyes with a low LLIT than in eyes with a high LLIT. From an artistic point of view these findings imply that capturing the faint scleral arc of sclerotic scatter (Fig 2) requires proportionally less lateral light in photographs or classical paintings shot or painted under mesopic conditions than under photopic conditions. Under photopic conditions, the probability of capturing the arc depends partly on the variable and unpredictable efficiency of the subject's eye in transmitting lateral light.

At low illuminance levels (≤ 40 lux: mesopic conditions), our censored linear model shows that sclerotic scatter perception is triggered by illuminance levels of incident light 34.4 times greater than those of ambient light. This figure is to be taken with caution, the likelihood of the censored linear model being far inferior to that of the log-log model. Still, this finding is consistent with slit-lamp biomicroscopy recommendations to use a very bright slit beam to illuminate the limbus when performing sclerotic scatter [2,4], or to use a "bright torch" to illuminate the limbus in order to trigger sclerotic scatter in the opposite non-illuminated limbus [7]. In sclerotic scatter, the cornea behaves like a solid curved volume that guides light by means of total internal reflection from one limbus to the other in a similar way to that originally described in 1842 by the Swiss physicist Jean-Daniel Colladon for a parabolic liquid (water) stream [14]. Sclerotic scatter is usually used to qualify the biomicroscopic technique aimed at spotting and mapping corneal lesions [1–5]. However, strictly speaking, sclerotic scatter refers to a dual phenomenon: scattering of incident light by the limbal sclera on one side and scattering of the light guided by the cornea to the limbal sclera on the other side where it creates a limbal arc of light (Figs 1 and 2). Ayoub and Said used the term "scleral scatter" to denote this limbal arc of light phenomenon [7]. In the present study, "sclerotic scatter" does not refer to the biomicroscopic examination technique [1] but solely to the scattering of light on the illuminated limbal sclera, then on the opposite limbal sclera (after the light has travelled through total internal reflection into the cornea) where it forms a limbal arc of light. In sclerotic scatter, the incident light is scattered in no particular direction–as has only ever been clearly described and illustrated in Graves' original work [1] in comparison with other sources we consulted [2–4,7,15–17]–and a small fraction of it is therefore expected to enter the limbal cornea [1]. The refractive indices of air, the cornea, and aqueous humor are 1, 1.376 and 1.336 respectively [18]. Therefore, the critical angles—i.e. the angle between the light ray and the tangent below which the light ray undergoes total internal reflection [18]–for the air-cornea and cornea aqueous-humor interfaces are 48.4° ($90-\left({\text{sin}}^{-1}\frac{1}{1.376}\right)\frac{180}{Pi}$) and 13.8° ($90-\left({\text{sin}}^{-1}\frac{1.336}{1.376}\right)\frac{180}{Pi}$) respectively. Given this low angle value for the cornea-aqueous humor interface, a fraction of the entering light is expected to reach the other side, where the reflected light undergoes sclerotic scatter a second time [1,4]. Once again, the scattering occurs in no particular direction (Fig 1), and only the light scattered towards the examiner's eye may be seen. Overall, a tiny fraction of the incident light on the limbus is therefore expected to be seen, a fact that is consistent with our findings that, at low illuminance levels (≤ 40 lux), ALI and the LLIT are linearly related with a high coefficient of 34.4.

In the present study, the measurement error was evaluated as a ratio and bore no particular relationship with the ALI. Moreover, as ALI values increased, LLIT values increased up to more than 10 000 lux, i.e. the lighting level of a bright cloudy day [19]. Hence, during the experiments for high photopic ALI values, the application of light to the temporal limbus resulted in a very bright spot reflected by the sclera. It may be that in some instances, this bright spot attracted the eyes of the examiners [20] and resulted in an afterimage that disturbed the examiners' ability [20] to spot the limbal crescent. The high dispersion of LLIT values for high ALI levels may thus partly be explained. Regarding the issue of why proportionally higher LLIT values are required for higher ALI values, one should keep in mind that the sclerotic scatter limbal arc elicited in this study depended on the two human examiners' perception. In this regard, it should be mentioned that Paulson and Sjöstrand tried to measure the decrease in contrast sensitivity when a bright light source was introduced into the visual field [21]. They showed that in healthy volunteers, the decrease in contrast sensitivity in the presence of a glare light was most distinct at low and medium frequencies [21]. Discrimination of the rather thick sclerotic scatter limbal arc requires mostly low contrast (i.e. contrasts with low spatial frequency) perception. For high ALI levels, the high LLIT levels required to elicit the sclerotic scatter limbal crescent resulted in an increasingly brighter light spot, the result of which may have been to decrease low-contrast sensitivity [21], thereby inducing proportionally higher LLIT values for photopic rather than mesopic ALI values. This phenomenon, which makes the perception of the limbal arc of light less clear-cut, may also partly account for the high dispersion of LLIT values for high ALI levels.

Our study results did not substantiate our first impression that subjects with a light iris shade had lower LLIT. This finding suggests that total internal reflection within the cornea is by far the most important light pathway to reach the opposite limbus, as described by Graves [1]. The other pathways all involve reflection on the iris (see Fig 1) and include refraction followed by reflection on the iris [3,22]; sclerotic scatter followed by reflection on the iris and by total internal reflection within the anterior chamber [23]; and sclerotic scatter followed by refraction through the cornea-aqueous humor interface then reflection on the iris. Such pathways could possibly have played a role, which prompted our decision to use an 8 mm spot straddling the limbus (as opposed to a slit beam directed exclusively onto the limbus) to induce iris illumination by refracted light [3]. However, our study shows that these pathways play no significant role. Indeed, we found no statistically significant difference between light and dark iris shades, the latter being expected to absorb more light [22] and increase the LLIT.

The present study showed an influence of the mean keratometry values on LLIT, an effect which was counter-balanced by the interaction of mean keratometry with ALI. Though statistically significant, the influence of the mean keratometry values and of the interaction of mean keratometry with ALI on the LLIT was very limited. The absolute value of the coefficient of these parameters were respectively 15 and 68 times smaller than the coefficient attributed to the most important parameter, i.e. the logarithm of the ALI. For this reason, the statistical significance of keratometry should not be overinterpreted. We believe that trying to substantiate these finding through modelization would be difficult, given the complex nature of sclerotic scatter [1] and of the human cornea, an aspherical diopter of known complexity [24]. The focusing of postero-temporal peripheral light by the cornea through the anterior chamber to the nasal limbus [25,26]–also known as the "Coroneo effect" [27]–has been reported to occur more frequently [25] and with higher intensity [26] in subjects with higher keratometric values. In such subjects, our study shows that under low ALI values, slightly less light scatter occurs at the nasal limbus for a given temporal light illuminance. A slightly darker scleral arc is expected to ensue. It might be that this darker scleral arc provides a background that makes the postero-temporal light focused by the cornea through the anterior chamber to the nasal limbus [27] more visible.

Central corneal pachymetry did not correlate with LLIT values. It may be that central corneal pachymetry does not capture enough of the complex human corneal morphology [24] to account for such an intricate phenomenon as sclerotic scatter.

One of the limitations of this study is the absence of control of ALI during application of the lateral light beam. This beam was partially reflected in all directions by the temporal sclera, which may have increased the ALI. However, the zone under scrutiny (the nasal limbus) was opposite the light beam, relatively protected from the reflected light. This could have become a substantial confounding factor for a lateral light source (90° from the sagittal plane) and explains why we chose a lateral light source located 30° from the sagittal plane. Another limitation was the absence of control of dark adaptation during our protocol. Indeed, during the experiment the LLIT was determined immediately after having set the ALI to a given level, leaving almost no time for dark adaptation to occur [28].

From a clinical point of view, the posterior margin of the limbal light arc characteristic of "sclerotic scatter" (as defined in the introduction) is projected in front of the supraciliary space, 0.5 mm behind the scleral spur [7]. This finding may be helpful in the operating room. Using either an surgical microscope with a slitlamp attachment [29] or a light-pipe, one can apply light to a part of the limbus and make the light scleral arc appear on the opposite side. In this study, we have found, that perception of this arc requires extreme contrast between the ambient light (ALI) and the lateral light (LLIT) striking one limbus and that overall, this contrast increases as ALI increases. Accordingly, the operating room should be kept as dark as possible in order to facilitate the limbal scleral arc perception. Diode laser cycloablation is a procedure in which proper positioning of the probe is front of the pars plicata ciliaris is paramount [30]. By positioning the anterior part of the probe tangent to the posterior part of the light arc (i.e. 0.5 mm behind the scleral spur), one ensures delivering the laser energy to the pars plicata ciliaris. Clearly spotting the light arc may also be helpful in several glaucoma procedures as first described by Ayoub and Said in 1973. More specifically, spotting the posterior part of the scleral arc may help find the scleral spur, an anatomical landmark of utmost importance for suprachoroidal T-flux implantation [31] or deep sclerectomy [32]. If the sclerotic scatter limbal arc is for operating purposes (to find the scleral spur or to place the probe for diode cycloablation), extreme contrast is required, and the present study shows that this is most consistently achieved under mesopic conditions.

In conclusion, this study shows that sclerotic scatter perception requires extreme contrast between the ambient light (denoted by ALI) and the lateral light (denoted by the LLIT) striking one limbus while leaving the opposite limbus in the shade. Overall, this contrast increases as ALI increases. Under mesopic conditions (defined as ALI values of 0.05 lux to 50 lux [8,9]) sclerotic scatter is consistently triggered by LLIT values that are around 34 times greater than the ALI value. In general, but notably more so under photopic conditions (defined as ALI values superior to 50 lux [8,9]) than under mesopic conditions, the LLIT values required to trigger sclerotic scatter are proportionally greater and more dispersed for higher values of ALI than for lower values.
